# Prenatal Exposure to Source-Specific Fine Particulate Matter and Autism Spectrum Disorder

**DOI:** 10.1021/acs.est.4c05563

**Published:** 2024-10-11

**Authors:** David G. Luglio, Michael J. Kleeman, Xin Yu, Jane C. Lin, Ting Chow, Mayra P. Martinez, Zhanghua Chen, Jiu-Chiuan Chen, Sandrah Proctor Eckel, Joel Schwartz, Frederick Lurmann, Rob McConnell, Anny H. Xiang, Md Mostafijur Rahman

**Affiliations:** † Department of Environmental Health Sciences, Tulane University School of Public Health and Tropical Medicine, New Orleans, Louisiana 70118, United States; ‡ Department of Civil and Environmental Engineering, University of California, Davis, Davis, California 95616, United States; § Spatial Science Institute, University of Southern California, Los Angeles, California 90089, United States; ∥ Department of Research & Evaluation, Kaiser Permanente Southern California, Pasadena, California 91101, United States; ⊥ Department of Population and Public Health Sciences, Keck School of Medicine, University of Southern California, Los Angeles, California 90089, United States; # Department of Environmental Health, Harvard T.H. Chan School of Public Health, Boston, Massachusetts 02115, United States; ∇ Department of Epidemiology, Harvard T.H. Chan School of Public Health, Boston, Massachusetts 02115, United States; ○ Sonoma Technology, Inc., Petaluma, California 94954, United States

**Keywords:** autism spectrum disorders, PM2.5, gasoline, pregnancy, air pollution sources, prenatal exposures

## Abstract

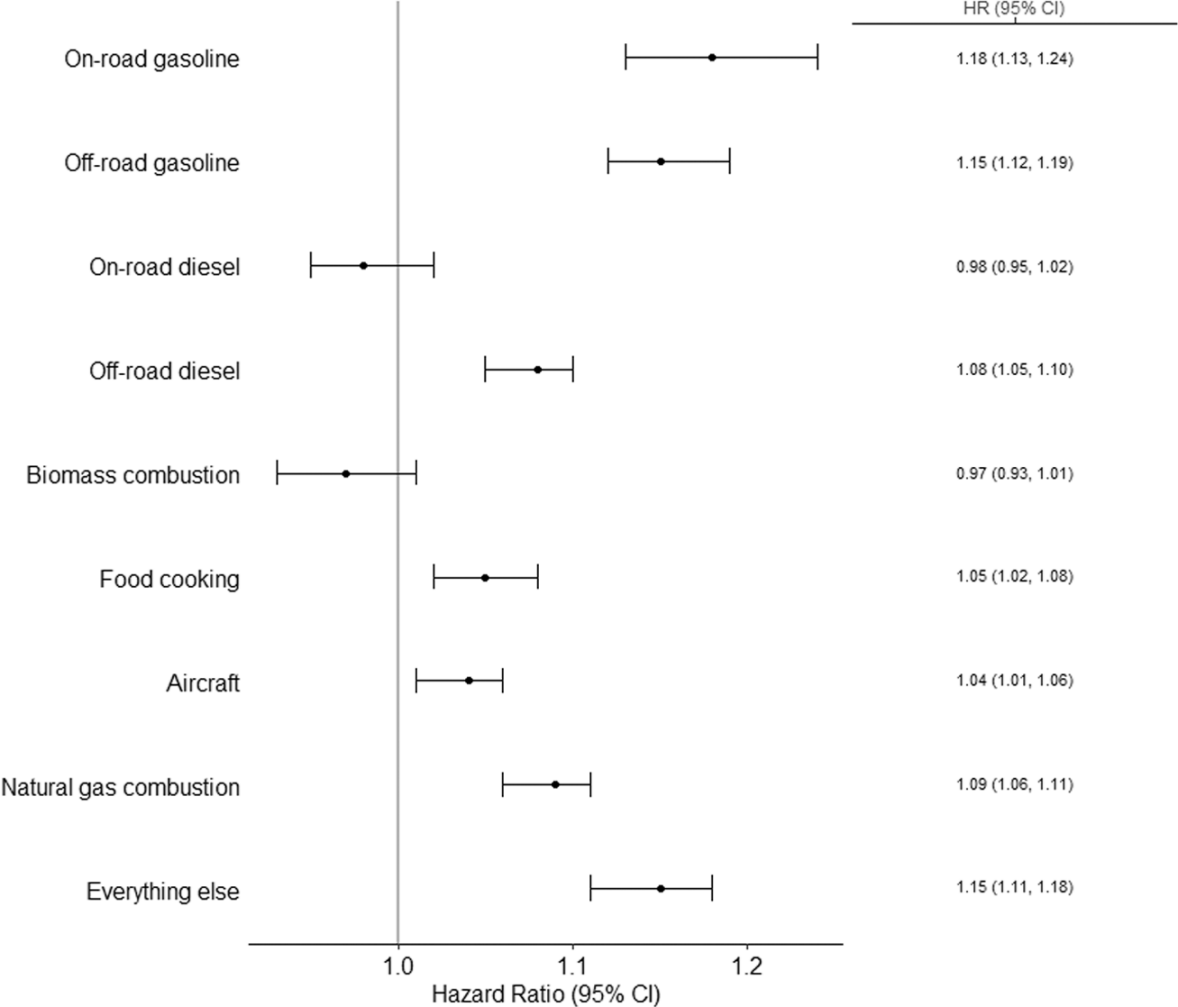

In this study, associations between prenatal exposure to fine particulate matter (PM2.5) from 9 sources and development of autism spectrum disorder (ASD) were assessed in a population-based retrospective pregnancy cohort in southern California. The cohort included 318,750 mother–child singleton pairs. ASD cases (*N* = 4559) were identified by ICD codes. Source-specific PM2.5 concentrations were estimated from a chemical transport model with a 4 × 4 km^2^ resolution and assigned to maternal pregnancy residential addresses. Cox proportional hazard models were used to estimate the hazard ratios (HR) of ASD development for each individual source. We also adjusted for total PM2.5 mass and in a separate model for all other sources simultaneously. Increased ASD risk was observed with on-road gasoline (HR [CI]: 1.18 [1.13, 1.24]), off-road gasoline (1.15 [1.12, 1.19]), off-road diesel (1.08 [1.05, 1.10]), food cooking (1.05 [1.02, 1.08]), aircraft (1.04 [1.01, 1.06]), and natural gas combustion (1.09 [1.06, 1.11]), each scaled to standard deviation increases in concentration. On-road gasoline and off-road gasoline were robust for other pollutant groups. PM2.5 emitted from different sources may have different impacts on ASD. The results also identify PM source mixtures for toxicological investigations that may provide evidence for future public health policies.

## Abstract 2

This study reports associations between prenatal PM2.5 exposures from specific sources on autism spectrum disorder development. It builds on previous work examining air pollution effects on autism.

## Introduction

Autism spectrum disorder (ASD) is a set of social communicative and behavioral challenges related to atypical neurodevelopment. These characteristics range from repetitive behaviors and patterns to difficulty interacting with others and even an inability to talk.^1−3^ ASD is diagnosed at an early age and persists throughout adulthood;^4−6^ therefore, ASD etiologic research focuses mainly on prenatal exposures.

Prior research has primarily focused on genetic risk factors for ASD, identifying over 100 genes associated with ASD. These genes account for a large fraction of ASD variation.^7,8^ Recent literature, additionally, has revealed associations between several environmental exposures and increased risk of ASD. Prenatal exposure to air pollution, most consistently to fine particulate matter (PM2.5), has been associated with ASD among children.^9−20^ Other associated pollutants were ozone (O_3_) and nitrogen dioxide (NO_2_).^9,13,17^ PM2.5 is composed of different species.^21−25^

PM2.5 is regulated based on total concentration, but studies indicate that different compositions have different effects on several health outcomes.^26−31^ We recently reported PM2.5-composition-specific associations, such as black carbon (BC), organic matter (OM), nitrate (NO_3_^–^), and sulfate (SO_4_^2–^), on ASD.^15^ In another study, we reported strong association of ASD with components related to nontailpipe emissions, such as copper (Cu), iron (Fe), and manganese (Mn).^16^

New models of spatial variability in sources of PM2.5 are relatively recently available. To the best of our knowledge, only a single study from Sweden examined the effect of different PM sources on ASD.^32^ Identification of the risk associated with increased exposure to sources of PM would have important implications. Different sources of PM may emit different mixtures of components or, at times, uniquely prominent components or forms of components (e.g., different oxides of the same metal). Given this, it may be expected that different PM2.5 sources have different toxicities, which would have bearings on emergent outcomes, such as ASD development. In studies of other outcomes, PM2.5 components derived from fossil-fuel combustion are consistently found to be the most toxic. Therefore, it has been proposed that regulation of PM2.5 should be tailored to the component,^33^ although there are arguments against this given the mixed findings about the relative potencies of different components and challenges associated with operationalizing source-specific regulations.^34−36^ Here, we focus on sources relevant to the ASD.

Our aim of this study was to assess the differential effects of prenatal exposure to ambient PM2.5 sources at maternal addresses on ASD development among children in a large population-based southern California pregnancy cohort. In this cohort, we have previously reported associations of ASD with aircraft-sourced PM2.5, using the same exposure modeling approach we use in this study.^10^ We now examined the associations of eight other sources with ASD. We also assessed whether the source-specific exposure associations were independent of each other and of associations with PM2.5 mass.

## Materials and Methods

### Study Population

We used a large, multiethnic population-based retrospective pregnancy cohort study to address the study questions. The cohort included mother–child pairs of singletons delivered at Kaiser Permanente Southern California (KPSC) hospitals between January 1, 2001 and December 31, 2014. KPSC is a large integrated healthcare system with over 4.5 million members and is representative, demographically, of the population of the region.^37^ Information on maternal social and demographic characteristics, as well as pregnancy health information, was extracted from KPSC’s electronic medical records (EMR) system. Maternal addresses during pregnancy were also extracted from the EMR and were geocoded using ArcGIS. We further assessed the geocodes for exposure assignment suitability. Mother–child pairs with addresses with only a street name, locality, administrative unit, or 5-digit postal code were excluded from the study since they provided a location too uncertain for exposure assignment.

The starting cohort size used in this study was 370,723 maternal–child pairs, inclusive of singleton births with continued KPSC membership at age 1. A total of 51,973 births were excluded, including those with implausible age of delivery or birth weight and missing covariates (*n* = 666), out-of-range maternal age of delivery (i.e., age <15 years or >55 years; *n* = 159), and missing/incomplete addresses or geocodes not suitable for exposure assignment (*n* = 51,148). Detailed derivation of the sample size, including other exclusionary factors, is shown in Figure S1. The final cohort size was 318,750 mother–child pairs. Characteristics of this group are listed in Table 1. Screenings for potential ASD risk started at 18 months of life during well-child visits, and the median age of ASD diagnosis was 3 years. Children were followed from birth through EMR until the clinical diagnosis of ASD, death, loss to follow-up, or age 5, whichever came first. The follow-up period for this study concluded in December 2019, ensuring that children born in 2014 received a minimum of 5 years of follow-up time. To ensure uniform follow-up duration for all children, we applied censoring at the age of 5 years.

**Table 1:** Characteristics of Children, with and without Autism Spectrum Disorder (ASD)

	children, no. (%) or median (interquartile range)		
characteristics	overall (*n* = 318,750)	with ASD (*n* = 4559)	without ASD (*n* = 314,191)
sex			
male (%)	163 181 (51.2)	3703 (81.2)	159 428 (50.7)
female (%)	155 569 (49.8)	856 (18.8)	154 763 (49.3)
follow-up year after birth, median [IQR^e^ ], years	4.0 [4.0, 4.0.]	3.0 [2.3, 3.7]	4.0 [4.0, 4.0]
maternal age at delivery	30.4 [26.3, 34.3]	31.3 [27.5, 35.2]	30.4 [26.2, 34.3]
median [IQR^e^], years			
parity, *N* (%)			
0	111 981 (35.1)	1844 (40.4)	110 137 (35.1)
1	104 561 (32.8)	1495 (32.8)	103 066 (32.8)
>2	84 176 (26.4)	903 (19.8)	83 273 (26.5)
unknown	18 032 (5.7)	317 (7.0)	17 715 (5.6)
maternal education, *N* (%)			
high school or lower	112 096 (35.2)	1335 (29.3)	110 761 (35.3)
some college	94 524 (29.7)	1477 (32.4)	93 047 (29.6)
college graduate or higher	109 087 (34.2)	1713 (37.6)	107 374 (34.2)
unknown	3043 (1.0)	43 (0.7)	3009 (1.0)
household annual income,^a^*N* (%)			
<$30,000	24 027 (7.5)	325 (7.1)	23 710 (7.5)
$30,000–$49,999	100 575 (31.6)	1436 (31.5)	99 139 (31.6)
$50,000–$69,999	98 015 (30.7)	1415 (31.0)	96 593 (30.7)
$70,000–$89,999	55 611 (17.4)	801 (17.5)	54 816 (17.4)
>$90,000	40 512 (12.7)	582 (12.8)	39 933 (12.7)
race/ethnicity, *N* (%)			
non-Hispanic white	81 050 (25.4)	956 (21.0)	80 094 (25.5)
non-Hispanic black	29 773 (9.3)	477 (9.8)	29 326 (9.3)
Hispanic	161 414 (50.6)	2300 (50.4)	159 114 (50.6)
Asian/Pacific Islander	39 974 (12.5)	744 (16.3)	39 230 (12.5)
other	6539 (2.1)	112 (2.5)	6427 (2.0)
any history of maternal comorbidity,^b^*N* (%)	46 717 (14.6)	839 (18.4)	45 878 (14.6)
prepregnancy diabetes,^c^*N* (%)	10 248 (3.2)	242 (5.3)	10 006 (3.2)
prepregnancy obesity,^d^*N* (%)	53 354 (16.7)	1049 (23.0)	52 305 (16.6)
year of birth, *N* (%)			
2001–2007	152 750 (47.9)	1802 (39.5)	164 198 (52.2)
2008–2014	166 000 (52.1)	2757 (60.5)	149 993 (47.2)

a Census tract-level median household income.

b ≥1 diagnosis of heart, lung, kidney, or liver disease; cancer.

c Type I and Type II diabetes diagnosed before pregnancy.

d Prepregnancy BMI ≥ 30.

e Abbreviations: IQR, interquartile range.

Both the KPSC and University of Southern California Institutional Review Board approved this study with a waiver of individual subject consent.

### ASD Ascertainment

ASD diagnoses were considered valid if identified by the ICD-9 codes 299.0, 299.1, 299.8, and 299.9 (for EMR records before October 1, 2015) and ICD-10 codes F84.0, F84.3, F84.5, F84.8, and F84.9 (after October 1, 2015) in at least two separate visits, as described in previous studies.^38−41^

### Air Pollution Exposure Assessment

A source-oriented chemical transport model developed by the University of California Davis/California Institute of Technology (UCD/CIT) was employed to assess prenatal source-specific PM2.5 exposures.^42^ This model tracked 22 PM constituents from emission through atmospheric transport and deposition, incorporating calculations on coagulation, gas- and particle-phase chemistry, and gas-to-particle conversion. Emission rates were derived from emission inventories provided by the California Air Resources Board (CARB), and meteorological data from the Weather Research and Forecast (WRF) model was incorporated to project particle chemical activity, movement, and fate. PM data were estimated in 3D atmospheric grid cells. The UCD/CIT CTM was configured to predict mass and number concentrations of particles ranging in diameter from 0.01 to 10 μm with 4 × 4 km^2^ horizontal grid resolution at the ground level in the current study. Results were generated with hourly time resolution but were averaged to monthly time resolution prior to use in the exposure analysis.^43,44^ The UCD/CIT model has been applied in multiple studies across the United States,^45^ but results from California are used in the current study.

PM concentrations emitted from 9 different source groups were tagged and tracked through the simulation of emissions, transport, and deposition. These source groups included on-road gasoline, on-road diesel, off-road gasoline, off-road diesel, natural gas combustion, food cooking, biomass burning, aircraft, and an all-other source category. We previously reported associations between aircraft-sourced PM2.5 and ASD development within this cohort,^10^ but not in the context of other sources. This current study builds on that work by adjusting for other specific-source PM2.5. Only primary particle contributions were tracked from each source group. Secondary coatings on particles were not tagged for source apportionment. Measured PM size and composition profiles were applied to PM emissions^46−51^ and followed through the atmospheric simulation. This model is based on updated work from ref (52) and is described in more detail in refs (43,44,53). Monthly averaged modeled concentrations were compared against all of the monthly averaged ambient monitoring data assembled by the EPA at all available locations and times. The bias between predicted and measured monthly average PM2.5 mass concentrations was used as a target for a constrained multilinear regression model based on the primary PM2.5 concentrations from on-road gasoline vehicles, off-road gasoline vehicles, on-road diesel vehicles, off-road diesel vehicles, biomass combustion, food cooking, aircraft, natural gas combustion, and all other sources. Additional independent variables were based on secondary nitrate and sulfate. Regression coefficients were constrained to range from ±5. Exposure concentrations were adjusted to remove bias at all locations across the CTM grid. Strong correlations (*r* > 0.8) were observed between the predicted and measured PM2.5 mass concentrations at most of the monitoring stations. Previous studies have compared the source-specific CTM predictions to measurements at receptor sites across California. Reasonable agreement between predictions and measurements is generally observed across all locations.^42^

Monthly average exposures to each of the sources and total PM2.5 mass were assigned to maternal addresses during the entire pregnancy. Monthly exposure estimates that did not correspond exactly to a pregnancy start/end month were assigned proportionally based on an overlap of the months. Exposures were time-weighted to account for changes in subject addresses during pregnancy.

### Covariates

Covariates were selected based on associations with ASD in previous studies and on expert knowledge.^39,54^ These variables included characteristics of the mother: self-reported race/ethnicity, age at delivery, parity, education levels, estimated household income based on census tract expressed as per 10k, and history of medical co-occurring conditions (e.g., ≥ diagnosis of heart, lung, kidney, or liver disease; cancer). Also included were child sex, birth year, and an indicator variable for season (i.e., dry from April to October; wet from November to March). ASD incidence has been observed to have increased over time, whereas PM concentrations have decreased. To control for this potential confounding time effect, models were adjusted for birth year. It was included as a nonlinear term with a penalized natural spline with four degrees of freedom as selected through the Akaike Information Criterion (AIC). Prepregnancy diabetes mellitus and obesity of the mother, but not birth weight or gestational age, were additionally included as covariates. The former two have been demonstrated risk factors for ASD,^41^ whereas the latter two were not adjusted for since they may be directly in the causal pathway.^55−57^ Finally, neighborhood socioeconomic status (SES) and urbanicity indicator variables were included in sensitivity analyses. The SES indicator was defined as the neighborhood disadvantage as in Yu et al.^58^ The urbanicity variable was classified into two levels (urban and suburban/rural) and assigned to each participant based on the USDA rural–urban commuting area codes based on the year 2000 census tracts and the mapping in a previous report.^59,60^

The total list of covariates is birth year, season, maternal age at delivery, maternal parity, maternal race/ethnicity, maternal education, maternal comorbidities (yes/no), maternal prepregnancy obesity (yes/no), maternal prepregnancy diabetes (yes/no), household income, and child gender.

### Statistical Analysis

Associations of pregnancy average exposure to PM from the 9 sources and the development of ASD were assessed using Cox proportional hazard models. A base model included all of the covariates described above in each analysis. Schoenfeld residual plots were constructed to assess whether the proportional hazards assumption was met, and no clear nonrandom pattern against follow-up time was discerned. Since the mean concentrations of PM varied from source to source, we standardized them by subtracting by their means and then dividing the values by their standard deviation when included in the model. Hazard ratios (HR) and 95% confidence intervals (CIs) associated with the ASD outcome were reported as a standard deviation (SD) increase in their respective source concentration so that the population HRs for each source were comparable relative to each other. A secondary analysis was conducted per 1 μg/m^3^ increase of source PM2.5 concentration to assess the inherent potency of each particle type. This was conducted only for those sources with significant positive associations in the population-relevant analysis. We first fitted the single-source model. In sensitivity analyses, to assess the independence of PM2.5 source association, we adjusted the single-source model with total PM2.5 mass, as well as remainder PM2.5 mass (i.e., PM2.5 mass minus source-specific mass), as described previously.^15^ The correlations between sources were not exceptionally high (see Table 3); therefore, we additionally ran a multisource model including all sources simultaneously to assess the relative strength of the association of each source. Each of the covariates listed above were included into each of the models. In a previous study, SES (defined as neighborhood disadvantage) was found to be associated with ASD^58^ in this cohort but was not included in the current main model since related variables such as income, race/ethnicity, and maternal education were already included. Additionally, prior work has found that autism diagnosis rates vary by urbanicity with higher numbers in urban areas;^61,62^ therefore, sensitivity analyses were conducted with inclusion of these two variables separately and simultaneously to test whether the results of our primary analyses are robust to these adjustments. Since 45,680 participants were excluded due to imprecise residential addresses (as mentioned above), which may lead to selection bias, we conducted an additional analysis including these excluded participants to assess if there was any change in the effect estimates. Robust standard errors were used to correct for potential correlations within siblings born to the same mothers. α value for statistical tests was set at 0.05. All analyses were performed using R Statistical Software (v4.1.2).^63^

## Results

A total of 4559 children were diagnosed with ASD by the age of 5, with males making up more than 80% of the cases/diagnoses (Table 1). The ASD and non-ASD groups showed small differences in maternal age at delivery and household income distributions. Furthermore, children with ASD were more likely to have mothers with co-occurring medical conditions, as well as prepregnancy diabetes and obesity. In addition, these mothers were more often nulliparous.

**Figure 1:**
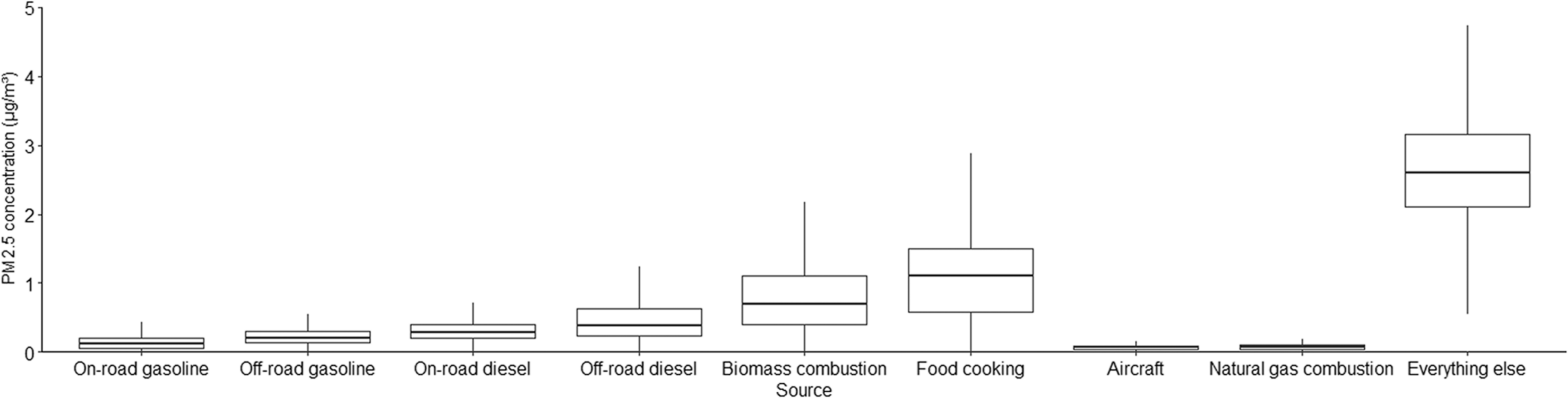
Pregnancy-averaged source-specific PM_2.5_ exposure concentrations across participants (outliers removed). Outliers were selected as data points 1.5 × IQR greater than the third quartile.

Table 2 shows the pregnancy average and median exposures to PM2.5 concentrations, in total and by source. Note that the all-source PM2.5 is a summation of all the sources, whereas the total PM2.5 concentration is the estimated measurement by the model as a group. This latter term includes secondary particles that are excluded from each of the source groups. The largest identified air pollution source affecting this population was food cooking and biomass combustion with average exposures of 1117.8 and 858.1 ng/m^3^, respectively. The pregnancy averages of other common sources such as on-road gasoline, on-road diesel, and natural gas combustion were 148.9, 315.1, and 80.8 ng/m^3^, respectively. Since the UCD/CIT model only tags primary PM emissions, each of these sources composes a small percentage of total PM2.5 mass. The variability in concentrations in each of the sources differed, with the largest range observed in the “other”/“everything else” and food cooking categories. The typical spread of exposures is displayed in Figure 1, excluding all outliers (i.e., data points than 1.5 × IQR greater than the third quartile), which are shown in Figure S2. Notably, the peak exposures are to biomass combustion particles despite having a lower overall median or mean than food cooking or the “other”/“everything else” categories. Sources in the other category include windblown dust and tire and brake wear. SD increases were highest proportionally for aircraft (coefficient of variation [CV]: 1.14) and lowest proportionally for “other”/“everything else” (0.31). Spatial distribution patterns for different sources estimated by this model are shown by Yu et al.^42^

**Table 2:** Descriptive Statistics of PM_2.5_ Concentrations According to the Source of Emission^a^

	PM_2.5_ concentration (ng/m^3^)			
sources	mean	SD	median	IQR
on-road gasoline	148.9	135.1	115.0	151.3
off-road gasoline	232.8	120.9	204.1	161.4
on-road diesel	315.1	161.5	285.1	205.4
off-road diesel	473.0	346.2	385.7	395.0
biomass combustion	858.1	779.1	704.2	718.6
food cooking	1117.8	626.7	1101.5	920.7
aircraft	83.9	95.4	66.8	42.8
natural gas combustion	80.8	51.4	75.0	56.0
other/everything else	2659.8	811.5	2610	1050.4
all-source PM_2.5_ mass	5970.2	2106.2	5850.7	2824.9
total PM_2.5_ mass	14242.1	4097.8	13575	5459.3

a Abbreviations: SD, standard deviation; IQR, interquartile range.

There were only two instances of high correlations between source and total PM2.5 (*r* > 0.8), e.g., on-road gasoline and off-road diesel. Correlations among other sources were moderate, all less than 0.8. The highest correlation was 0.77 between the natural gas combustion and food cooking (Table 3).

**Table 3:** Spearman Rank Correlations between PM_2.5_ Sources and Total Mass

	on-road gasoline	off-road gasoline	on-road diesel	off-road diesel	biomass combustion	food cooking	aircraft	natural gas combustion	other	total PM_2.5_ mass
on-road gasoline	1.00	0.54	0.31	0.74	0.51	0.44	0.22	0.54	0.68	0.83
off-road gasoline		1.00	0.24	0.74	0.53	0.46	0.21	0.47	0.72	0.62
on-road diesel			1.00	0.41	0.06	0.70	0.33	0.72	0.52	0.29
off-road diesel				1.00	0.55	0.55	0.17	0.65	0.76	0.82
biomass combustion					1.00	0.18	0.13	0.19	0.39	0.65
food cooking						1.00	0.15	0.77	0.54	0.37
aircraft							1.00	0.33	0.46	0.20
natural gas combustion								1.00	0.75	0.48
other									1.00	0.72
total PM_2.5_ mass										1.00

**Figure 2:**
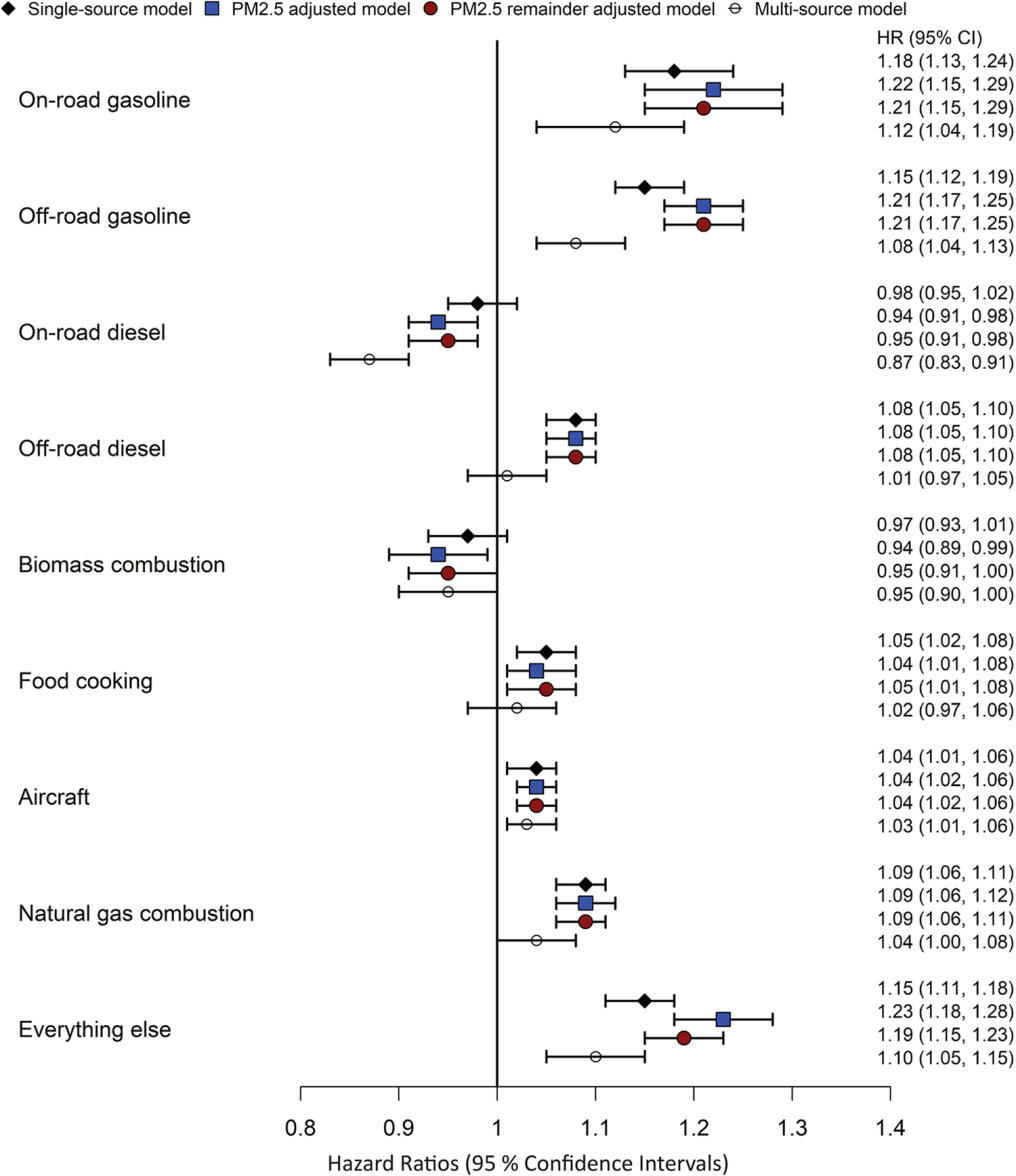
Hazard ratios (HR) of ASD scaled to standard deviation increases in each of the 9 source categories, estimated in single-, PM_2.5_ adjusted-, and multipollutant models. Standard deviations for each of the sources are given in Table 2.

In the single-source model analysis for the associations with ASD, pregnancy exposure to all sources, except for on-road diesel and biomass combustion, were significantly associated with increased risk of ASD (Figure 2). The HRs [95% CI] were highest for on-road gasoline (1.18 [1.13, 1.24]; per 135.1 ng/m^3^), off-road gasoline (1.15 [1.12, 1.19]; per 120.9 ng/m^3^), and “other”/“everything else” (1.15 [1.11, 1.18]; per 811.5 ng/m^3^). HRs for the other significant sources include off-road diesel (1.08 [1.05, 1.10]; per 346.2 ng/m^3^), food cooking (1.05 [1.02, 1.08]; per 626.7 ng/m^3^), natural gas combustion (1.09 [1.06, 1.11]; per 51.4 ng/m^3^), and previously reported aircraft-sourced PM2.5 (1.04 [1.01, 1.06]; per 95.4 ng/m^3^). These source-specific associations of increased ASD risks remained largely unchanged when adjusting the single-source model for the total PM2.5 and remainder PM2.5 mass. Notably, however, on-road diesel goes from a null association in the single-source model to a protective one in the PM2.5 adjusted and multisource models. The HR for the total and remainder PM2.5 variables are listed by the source model in Table S1. The HR for on-road gasoline, off-road diesel, food cooking, and natural gas combustion models were inclusive of 1.00. Furthermore, total or remainder PM2.5 had an inverse association with ASD when included in the off-road gasoline and “other”/“everything else” source models. In contrast, total and remainder PM2.5 maintained a significant association with ASD development in on-road diesel, biomass combustion, and aircraft-source models. Note that on-road diesel and biomass combustion exposures themselves had an inverse association or null association with the ASD in the PM2.5 adjusted models.

Including all 9 of the sources specified in this study in the same model (i.e., multipollutant model with different sources) resulted in noticeable attenuation of the HRs associated with some source-specific PM2.5 exposures (Figure 2). The association of ASD with on-road gasoline, off-road gasoline, aircraft, and “other”/“everything else” remained statistically significant; the associations of ASD with off-road diesel, food cooking, and natural gas combustion all weakened and lost statistical significance. The inverse association with on-road diesel became stronger and more significant. In the multisource model, the ASD HRs associated with on-road gasoline, off-road gasoline, aircraft, and “other”/”everything else were 1.12 (1.04, 1.19), 1.08 (1.04, 1.13), 1.03 (1.01, 1.06), and 1.10 (1.05, 1.15), respectively.

In the multisource model, the variance inflation factors (VIF) for each of the sources ranged from 1.16 to 4.77 (Table S2).

In sensitivity analyses, inclusion of SES and urbanicity factors did not materially change the effects estimates (Table S3) nor did analyses that included subjects with imprecise addresses excluded from the study population (Table S4).

Associations between aircraft, on-road, and off-road gasoline (i.e., the sources with positive significant associations in the multisource model) and ASD development were also examined per 1 μg/m^3^ source concentration, which allows for comparisons of the potency of each aerosol type (Table S5). The HR results for these sources in a multisource model remain significant but are generally larger, as 1 μg/m^3^ was larger than common population-relevant (SD) exposures.

## Discussion

In this retrospective birth cohort of over 300,000 mother–child pairs in southern California, we found that prenatal exposures to PM_2.5_ concentrations from various sources were associated with an increased ASD risk among children. These sources include on-road and off-road gasoline, aircraft, off-road diesel, food cooking, and natural gas combustion. Among these, the associations with on-road gasoline, off-road gasoline, and (previously reported) aircraft-sourced PM2.5 remained significant in multipollutant models. Biomass combustion showed no associations, whereas on-road diesel showed an inverse effect in the multisource and PM2.5 adjusted models.

Prior studies using different exposure modeling approaches, reported association between near-roadway air pollution (NRAP) and ASD,^19^ but only for NRAP from nonfreeway sources.^11^ Nonfreeway vehicular traffic was overwhelmingly gasoline-powered during the period of the study, and on-road diesel was largely used in heavy-duty vehicles on freeways. Therefore, the observed increased risk from gasoline sources is consistent with those previous findings.

In the single-source models, off-road, but not on-road, diesel was strongly associated with the risk of ASD development. This could be due to off-road diesel sources being more likely to be located in closer proximity to residences compared with on-road diesel sources in this cohort. Source-specific exposures across southern California using the UCD/CIT CTM model showed concurrent on-road diesel and gasoline peaks across downtown Los Angeles.^42^ A secondary peak for the on-road diesel was found to the east of the city in an area notable for a heavy concentration of warehouses and heavy-duty truck traffic. It is unclear why the on-road diesel combustion particles had an inverse association with ASD development in the multisource model, as evidence is strong that diesel exhaust particulate is neurotoxic.^64,65^ Effects of multicollinearity is a potential reason for the discrepancy between the single-source and multisource models. In a previous study using exposure estimated from hazardous air pollutant (HAP) inventories, higher levels of diesel PM exposure, nondistinguishing between on- vs off-road sources, were associated with increased risk of ASD.^66^

Natural gas combustion and food cooking sources have not been previously shown to be associated with ASD, which we observed in all but the multisource model. A number of studies have examined the associations between natural gas production and other health outcomes;^67−70^ the exposures in most studies, however, are not limited to natural gas combustion directly but include emissions from diesel generators, drilling processes, and fugitive releases of various chemicals. In other studies, exposure specifically to gas flaring was associated with adverse health effects.^69,71^ Our study is the first to report the association of ASD (or any other child neurodevelopmental outcome) with prenatal exposure to natural gas combustion. Most studies on the effects of food cooking have focused on exposures in indoor settings, with inconsistent results across various health end points.^72−74^ We found no significant effects of either natural gas or food PM2.5 that were independent of the other sources (i.e., based on the results of the multisource model), although multicollinearity effects in the model could have attenuated the effect estimate and reduced the statistical significance. Aircraft-sourced PM, on the other hand, showed robust associations across all models. Associations of ultrafine aircraft PM and ASD were previously demonstrated in this cohort. These novel associations were described by Carter et al.^10^ We additionally showed that this association is robust to adjustment with other sources.

We did not see any positive association between prenatal exposure to biomass combustion-derived PM2.5 and ASD. Biomass combustion emission levels are driven mainly by wildfires, residential heating, and crop burning.^75−77^ A prior California study, which utilized PM metrics from the same UCD/CIT CTM, found no association between long-term exposure to biomass combustion PM and mortality in the California Teachers Study Cohort.^78^ Yet, a significant body of research found health effects of biomass combustion PM.^79−82^ This includes a recent study in Sweden, which found increased ASD risk in association with residential wood burning.^32^ Therefore, the absence of an association between biomass PM derived from the UCD/CIT CTM with ASD in this study, and with mortality previously, could stem from the model’s inability to adequately represent the plume rise of wildfires or the sharp spatial gradients associated with residential wood combustion.

The “everything else”/“other” category, which was the largest single contributor to total PM2.5 mass, had a strong association with ASD development across all models. This category includes windblown dust, construction dust, tire wear, brake wear, and other types of fugitive dust emissions. The Swedish study related exposure to a smaller set of sources to ASD diagnosis.^32^ It was found that small-scale residential heating, tailpipe exhaust, and vehicle wear-and-tear were all associated with ASD risk. The traffic-related associations match well with our results and with previous work examining association with near-roadway air pollution and constituents of tailpipe and nontailpipe emissions from roadways in this cohort using different exposure modeling strategies.^11,16^ In those studies, tailpipe combustion tracers black and elemental carbons (BC and EC) had significant positive associations with ASD. Moreover, nontailpipe tracers Cu, Fe, and Mn also showed positive associations consistent with the results from the Swedish source study. An earlier study of the effects of PM components in this cohort also found associations of EC/BC and organic matter with ASD.^15^ A few other studies have examined ASD association with several air toxics.^66,83,84^

In our study, gasoline, both on- and off-road, and aircraft were robustly associated with ASD. The effect sizes were based on population-relevant exposures (SD) specific to each source. To evaluate the potency of the particles for each of these three sources, HR results were examined per 1 μg/m^3^ increase in PM2.5 concentration (Table S2). The variability in effect size indicates that potency (per 1 μg/m^3^) was larger for on-road gasoline and the least for aircraft particles. Nevertheless, these HR values are not substantially different from one another, suggesting that the potency of the particles was not markedly different.

There are numerous studies evaluating the toxicology of PM from various sources. Previous work has evaluated the effects of gasoline and aircraft particles in lung epithelial cells,^85−89^ with evidence for cytotoxicity, oxidative stress, and alteration of cytokine production. Studies have focused on toxicological comparisons across different vehicle fuel types, namely, between gasoline, diesel, and biofuels.^90−92^ One in China reported greater cytotoxicity and oxidative stress potential in gasoline vs diesel combustion particles.^92^ Comparative studies have also examined other sets of sources.^93−96^ For example, Karlsson et al.^96^ compared DNA damage and cytokine production in lung epithelial cells exposed to wood combustion, tire and road wear, subway, and street-side particles. The strongest cytokine responses were driven by the street particles and DNA damage by the subway particles.

Total PM2.5 mass by itself was associated with ASD, consistent with previous findings from this cohort.^15−17,39^ When coadjusted with some source-specific PM2.5, however, both total PM2.5 and remainder PM2.5 had generally smaller coefficients of effect. This occurred when PM2.5 was coadjusted for those sources with markedly large effects (e.g., on-road gasoline), but not for those with weaker effects (e.g., aircraft, biomass combustion, on-road diesel). Surprisingly, total and remainder PM2.5 were inversely associated with ASD risk when adjusted for off-road gasoline and the “other”/“everything else” sources. Protective effects are not biologically plausible given robust toxicological and epidemiological evidence on neurodevelopmental toxicity, including on ASD. Collinearity of the source PM with the total (and remainder) PM2.5 may explain the loss of significance in the coadjusted models. On-road gasoline and off-road diesel PM2.5 had correlation coefficients with total PM2.5 of 0.83 and 0.82, respectively. However, the variation inflation factors (VIF) for each source in the multisource model (Table S2) were not remarkably high (less than 5). Further investigation is warranted.

This study assessed associations of ASD with primary source-specific particulate matter (total PM2.5 contained secondary PM2.5 as well). Secondary particulate matter forms in the atmosphere from the reaction of gaseous precursor species, which can be emitted from many sources. Hypotheses about the health effects of primary particulate matter are easier to test, and the sources that are associated with negative health effects are potential targets for regulations. Hypotheses about the health effects of secondary particulate matter are more difficult to evaluate, and emissions controls to limit secondary particulate matter require a thorough understanding of formation mechanisms. A thorough evaluation of the sources and health effects of secondary particulate matter is beyond the scope of our study analysis.

A major strength of the study is the large and diverse cohort, representative of a major region of the United States.^37^ A wealth of demographic and health data are available from the established EMR system maintained by Kaiser Permanente. The CTM model has been employed in several epidemiological studies.^15,16,42,78,97−99^ This is a locally constructed and validated model used extensively in California and is likely to provide the most accurate and relevant results for this area. We were also able to adjust the estimated exposure to the residential movement of the subjects.

There are some limitations to the study. The CTM estimates outdoor ambient exposures for all locations; it does not estimate indoor exposures, which may be a significant contributor to total PM exposure. A lack of information on time spent in indoor and outdoor activities may have resulted in misclassification of the exposure estimate. However, unless there were consistent patterns of time–activity or indoor exposures that were different in the ASD and non-ASD groups during pregnancy, it is unlikely that missing this information would bias the effects differentially. PM2.5 source data used in this analysis were available at 4 km spatial resolution, which limited the ability to assess fine-scale variability of sources. The consequence of this exposure misclassification for epidemiological investigations due to coarser spatial resolution is likely to underestimate effects because the misclassification will likely be nondifferential to the outcome. Finally, there were additional covariates for which we have not adjusted in the models. The consumption of folic acid has been related to ASD development^100^ but likely has no association with air pollution levels. Detailed dietary information was not available in the EMR. Furthermore, effects of maternal smoking have been previously assessed in this cohort, finding no association with ASD.^41^ Accordingly, smoking was left out of the analysis because it is an unlikely confounder. Outside work on this relationship has yielded mixed results.^101^ Other factors such as maternal medication use and the occurrence of various birth-related events (e.g., cesarean section) previously investigated^102,103^ were not assessed, as these variables may also be mediators instead of confounders.

Impacts of prenatal gasoline and aircraft PM exposure on ASD in this study provide targets for future toxicological and other research. A focus could be on how the individual components or the whole mixture of these sources affect prenatal neurodevelopment in toxicological and population studies. Our results are based on population-relevant exposures, demonstrating the public health importance of these exposures and effects with regulatory implications. Overall, these results are consistent with emerging evidence that combustion products from different sources have different biological effects. This suggests that regulation of PM2.5 might provide greater benefits if it is focused on reducing more toxic sources. However, there is strong evidence that PM2.5 mass, irrespective of components or sources, has health effects and an emerging body of literature that reductions in general PM2.5 result in improvements in a host of health outcomes.

In summary, this study provides evidence that the impacts of prenatal exposure to PM2.5 on ASD, an important neurodevelopmental outcome, vary by the source. The most consistent associations were observed for on- and off-road gasoline in southern California. Associations were also observed with food cooking and natural gas combustion in the single-source and PM2.5 adjusted models; however, these associations were weakened in the models adjusted for multiple sources. On-road diesel and biomass combustion were not associated with ASD. Results also identify PM source mixtures for toxicological investigations that may provide evidence for future public health policies.

## Data Availability

Dr. Xiang had full access to all of the data in the study. Drs. Luglio, Xiang, and Rahman take responsibility for the integrity of the data and accuracy of the data analysis.

## Supporting Information Available

Additional results in the forms of tables and figures, including sensitivity analysis and single figure showing the study sample derivation (PDF)

## Notes

**Role of the Funder/Sponsor** The funders had no role in the design and conduct of the study; collection, management, analysis, and interpretation of the data; preparation, review, or approval of the manuscript; and decision to submit the manuscript for publication.

## Notes

J.S. has testified on behalf of the U.S. Department of Justice in a case involving a Clean Air Act violation.

## Notes

**Data Sharing Statement** KPSC Institutional Review Board approved this study, with waiver of informed consent with the condition that raw data remain confidential and would not be shared. Thus, due to the sensitive nature of these data, the data are not available to be shared.
