# Supplementary material for: Prenatal Exposure to Source-Specific Fine Particulate Matter and Autism Spectrum Disorder
Source: Environ Sci Technol. 2024 Oct 11;58(42):18566–77. doi: 10.1021/acs.est.4c05563 (PMC11500427; doi:10.1021/acs.est.4c05563)
Supplement: Supplementary file 1 — es4c05563_si_001.pdf [file es4c05563_si_001.pdf]

Prenatal exposure to source specific fine particulate matter and autism spectrum disorder

David G. Luglio[1], Michael J. Kleeman[2], Xin Yu[3], Jane C. Lin[4], Ting Chow[4], Mayra P. Martinez[4], Zhanghua Chen[5], Jiu-Chiuan Chen[5], Sandrah Proctor Eckel[5], Joel Schwartz[6,7], Frederick Lurmann[8], Rob McConnell[5], Anny H. Xiang[4]*, Md Mostafijur Rahman[1,5]*

[1]Department of Environmental Health Sciences, Tulane University School of Public Health and Tropical Medicine, New Orleans, LA 70118, USA
[2]Department of Civil and Environmental Engineering, University of California, Davis, Davis, CA 95616, USA
[3]Spatial Science Institute, University of Southern California, Los Angeles, CA 90089, USA
[4]Department of Research & Evaluation, Kaiser Permanente Southern California, Pasadena, CA 91101, USA
[5]Department of Population and Public Health Sciences, Keck School of Medicine, University of Southern California, Los Angeles, CA 90089, USA
[6]Department of Environmental Health, Harvard T.H. Chan School of Public Health, Boston, MA 02115, USA
[7]Department of Epidemiology, Harvard T.H. Chan School of Public Health, Boston, MA 02115, USA
[8]Sonoma Technology, Inc., Petaluma, CA 94954, USA
*Md Mostafijur Rahman (mrahman8@tulane.edu) and Anny H. Xiang (anny.h.xiang@kp.org) contributed equally and serve as joint senior authors.

Summary: 10 pages, 5 tables, 2 figures

Table of Contents:

Table S1. HR for the PM2.5 covariates included in the source-specific models	S2
Table S2. Variation inflation index (VIF) for each of the sources contained in the multisource cox proportional hazard model	S3
Table S3. Sensitivity analysis with adjustment for a socio-economic status and urbanicity indicator variables.	S4
Table S4. Single source models analysis including the previously excluded portion of the cohort due to imprecise address	S7
Table S5. Hazard Ratios (HR) of ASD scaled to 1 μg/m^3 increases in three of the sources estimated in a multi-pollutant models adjusted for all sources (i.e., on-road and off-road gasoline, on-road and off-road diesel, biomass combustion, food cooking, aircraft, natural gas combustion, and “everything else”).	S8
Figure S1. Derivation of study sample.	S9
Figure S2. Pregnancy-averaged source-specific exposure concentrations across participants	S10

Table S1 – HR for the PM2.5 in models co-adjusted for source-specific exposure

	Source exposure	Source HR (95% CI)	PM2.5 HR (95% CI)
Total PM2.5	None	N/A	1.07 (1.01, 1.12)
	On-road gasoline	1.22 (1.15, 1.29)	0.95 (0.89, 1.01)
	Off-road gasoline	1.21 (1.17, 1.25)	0.87 (0.82, 0.93)
	On-road diesel	0.94 (0.91, 0.98)	1.13 (1.06, 1.20)
	Off-road diesel	1.08 (1.05, 1.10)	1.01 (0.95, 1.07)
	Biomass combustion	0.94 (0.89, 0.99)	1.11 (1.04, 1.18)
	Food cooking	1.04 (1.01, 1.08)	1.02 (0.96, 1.00)
	Aircraft	1.04 (1.02, 1.06)	1.07 (1.01, 1.13)
	Natural gas combustion	1.09 (1.06, 1.12)	0.99 (0.94, 1.05)
	"Other"/"Everything else"	1.23 (1.18, 1.28)	0.84 (0.78, 0.91)
Remainder PM2.5	On-road gasoline	1.21 (1.15, 1.29)	0.95 (0.89, 1.01)
	Off-road gasoline	1.21 (1.17, 1.25)	0.87 (0.82, 0.94)
	On-road diesel	0.95 (0.91, 0.98)	1.13 (1.06, 1.20)
	Off-road diesel	1.08 (1.05, 1.10)	1.01 (0.95, 1.07)
	Biomass combustion	0.95 (0.91, 1.00)	1.10 (1.04, 1.16)
	Food cooking	1.05 (1.01, 1.08)	1.02 (0.96, 1.08)
	Aircraft	1.04 (1.02, 1.06)	1.07 (1.01, 1.13)
	Natural gas combustion	1.09 (1.06, 1.11)	0.99 (0.93, 1.05)
	"Other"/"Everything else"	1.19 (1.05, 1.15)	0.86 (0.81, 0.92)

Table S2. Variation inflation index (VIF) for each of the sources contained in the multisource model

Source	VIF
On-road gasoline	4.73
Off-road gasoline	2.34
On-road diesel	2.23
Off-road diesel	2.14
Biomass combustion	1.96
Food cooking	2.46
Aircraft	1.17
Natural gas combustion	2.03
Everything else	2.81

Table S3. Sensitivity analysis with adjustment for a socio-economic status and urbanicity indicator variables.

		HR (95% CI)			
Source	Model	- SES - Urbanicity	+ SES	+ Urbanicity	+ SES + Urbanicity
On-road gasoline	Single source	1.18 (1.13, 1.24)	1.19 (1.13, 1.25)	1.19 (1.14, 1.25)	1.19 (1.14, 1.25)
	PM2.5-adjusted	1.22 (1.15, 1.29)	1.22 (1.15, 1.29)	1.22 (1.15, 1.29)	1.22 (1.15, 1.29)
	Remainder PM2.5-adjusted	1.21 (1.15, 1.29)	1.22 (1.15, 1.29)	1.22 (1.15, 1.29)	1.22 (1.15, 1.29)
	Multi-source	1.12 (1.04, 1.19)	1.12 (1.05, 1.20)	1.11 (1.04, 1.19)	1.11 (1.04, 1.19)
Off-road gasoline	Single source	1.15 (1.12, 1.19)	1.15 (1.12, 1.19)	1.16 (1.12, 1.19)	1.16 (1.24, 1.20)
	PM2.5-adjusted	1.21 (1.17, 1.25)	1.21 (1.17, 1.25)	1.21 (1.17, 1.26)	1.22 (1.15, 1.29)
	Remainder PM2.5-adjusted	1.21 (1.17, 1.25)	1.21 (1.17, 1.25)	1.21 (1.17, 1.25)	1.21 (1.17, 1.25)
	Multi-source	1.08 (1.04, 1.13)	1.08 (1.03, 1.13)	1.08 (1.04, 1.13)	1.11 (1.04, 1.19)
On-road diesel	Single source	0.98 (0.95, 1.02)	0.99 (0.95, 1.02)	0.99 (0.95, 1.02)	0.99 (0.96, 1.02)
	PM2.5-adjusted	0.94 (0.91, 0.98)	0.94 (0.91, 0.98)	0.94 (0.91, 0.98)	0.95 (0.91, 0.98)
	Remainder PM2.5-adjusted	0.95 (0.91, 0.98)	0.95 (0.91, 0.99)	0.95 (0.91, 0.99)	0.95 (0.91, 0.99)

	Multi-source	0.87 (0.83, 0.91)	0.87 (0.83, 0.91)	0.87 (0.83, 0.91)	0.87 (0.83, 0.91)
Off-road diesel	Single source	1.08 (1.05, 1.10)	1.08 (1.05, 1.11)	1.08 (1.06, 1.11)	1.08 (1.06, 1.11)
	PM2.5-adjusted	1.08 (1.05, 1.10)	1.08 (1.05, 1.11)	1.08 (1.05, 1.11)	1.08 (1.05, 1.11)
	Remainder PM2.5-adjusted	1.08 (1.05, 1.10)	1.08 (1.05, 1.11)	1.08 (1.05, 1.11)	1.08 (1.05, 1.11)
	Multi-source	1.01 (0.97, 1.05)	1.01 (0.97, 1.05)	1.01 (0.97, 1.05)	1.01 (0.97, 1.05)
Biomass combustion	Single source	0.97 (0.93, 1.01)	0.97 (0.93, 1.01)	0.97 (0.93, 1.01)	0.97 (0.93, 1.01)
	PM2.5-adjusted	0.94 (0.89, 0.99)	0.94 (0.89, 0.99)	0.93 (0.89, 0.99)	0.93 (0.89, 0.99)
	Remainder PM2.5-adjusted	0.95 (0.91, 1.00)	0.95 (0.91, 1.00)	0.96 (0.91, 1.00)	0.96 (0.91, 1.00)
	Multi-source	0.95 (0.90, 1.00)	0.95 (0.90, 1.00)	0.95 (0.90, 1.00)	0.95 (0.90, 1.00)
Food cooking	Single source	1.05 (1.02, 1.08)	1.05 (1.02, 1.09)	1.06 (1.02, 1.09)	1.06 (1.02, 1.09)
	PM2.5-adjusted	1.04 (1.01, 1.08)	1.05 (1.01, 1.09)	1.05 (1.01, 1.09)	1.05 (1.01, 1.09)
	Remainder PM2.5-adjusted	1.05 (1.01, 1.08)	1.05 (1.02, 1.08)	1.05 (1.02, 1.09)	1.05 (1.02, 1.09)
	Multi-source	1.02 (0.97, 1.06)	1.02 (0.97, 1.06)	1.02 (0.97, 1.06)	1.02 (0.97, 1.07)

Aircraft	Single source	1.04 (1.01, 1.06)	1.04 (1.01, 1.06)	1.04 (1.02, 1.06)	1.04 (1.02, 1.06)
	PM2.5-adjusted	1.04 (1.02, 1.06)	1.04 (1.02, 1.06)	1.04 (1.02, 1.06)	1.04 (1.02, 1.06)
	Remainder PM2.5-adjusted	1.04 (1.02, 1.06)	1.04 (1.02, 1.06)	1.04 (1.02, 1.07)	1.04 (1.02, 1.07)
	Multi-source	1.03 (1.01, 1.06)	1.03 (1.01, 1.06)	1.03 (1.01, 1.06)	1.03 (1.01, 1.06)
Natural gas combustion	Single source	1.09 (1.06, 1.11)	1.09 (1.06, 1.11)	1.09 (1.06, 1.11)	1.09 (1.07, 1.12)
	PM2.5-adjusted	1.09 (1.06, 1.12)	1.09 (1.06, 1.12)	1.09 (1.06, 1.12)	1.09 (1.07, 1.12)
	Remainder PM2.5-adjusted	1.09 (1.06, 1.11)	1.09 (1.06, 1.12)	1.09 (1.06, 1.12)	1.09 (1.07, 1.12)
	Multi-source	1.04 (1.00, 1.08)	1.04 (1.00, 1.08)	1.04 (1.00, 1.08)	1.04 (1.00, 1.08)
"Other"/"Everything else"	Single source	1.15 (1.11, 1.18)	1.15 (1.11, 1.19)	1.16 (1.12, 1.20)	1.16 (1.13, 1.20)
	PM2.5-adjusted	1.23 (1.18, 1.28)	1.23 (1.19, 1.29)	1.24 (1.19, 1.29)	1.24 (1.19, 1.29)
	Remainder PM2.5-adjusted	1.19 (1.15, 1.23)	1.19 (1.16, 1.23)	1.20 (1.16, 1.24)	1.20 (1.16, 1.24)
	Multi-source	1.10 (1.05, 1.15)	1.10 (1.05, 1.15)	1.11 (1.06, 1.16)	1.11 (1.06, 1.16)

Table S4. Single source models analysis including the previously excluded portion of the cohort due to imprecise address

	HR (95% CI)	
Source	- Excluded	+ Excluded
On-road gasoline	1.18 (1.13, 1.24)	1.19 (1.14, 1.25)
Off-road gasoline	1.15 (1.12, 1.19)	1.15 (1.12, 1.18)
On-road diesel	0.98 (0.95, 1.02)	0.98 (0.95, 1.01)
Off-road diesel	1.08 (1.05, 1.10)	1.08 (1.06, 1.11)
Biomass combustion	0.97 (0.93, 1.01)	0.97 (0.93, 1.01)
Food cooking	1.05 (1.02, 1.08)	1.05 (1.02, 1.08)
Aircraft	1.04 (1.01, 1.06)	1.04 (1.02, 1.06)
Natural gas combustion	1.09 (1.06, 1.11)	1.09 (1.05, 1.11)
"Other/everything else"	1.15 (1.11, 1.18)	1.15 (1.12, 1.19)

Table S5. Hazard Ratios (HR) of ASD scaled to 1 $\mu g/m^3$ increases in each of the three sources that were significant in multi-source models estimated in a multi-pollutant models adjusted for all sources (i.e., on-road and off-road gasoline, on-road and off-road diesel, biomass combustion, food cooking, aircraft, natural gas combustion, and "everything else").

Source	HR	lower CI	Upper CI
On-road gasoline	2.27	1.38	3.74
Off-road gasoline	1.89	1.34	2.68
Aircraft	1.37	1.06	1.78

Figure S1. Derivation of study sample.

*The sum of those with missing or error birth weight, gender, mother's race-ethnicity, and mother's age at delivery exceeds 666 because 40 children had missing or errors in 2 of these covariates

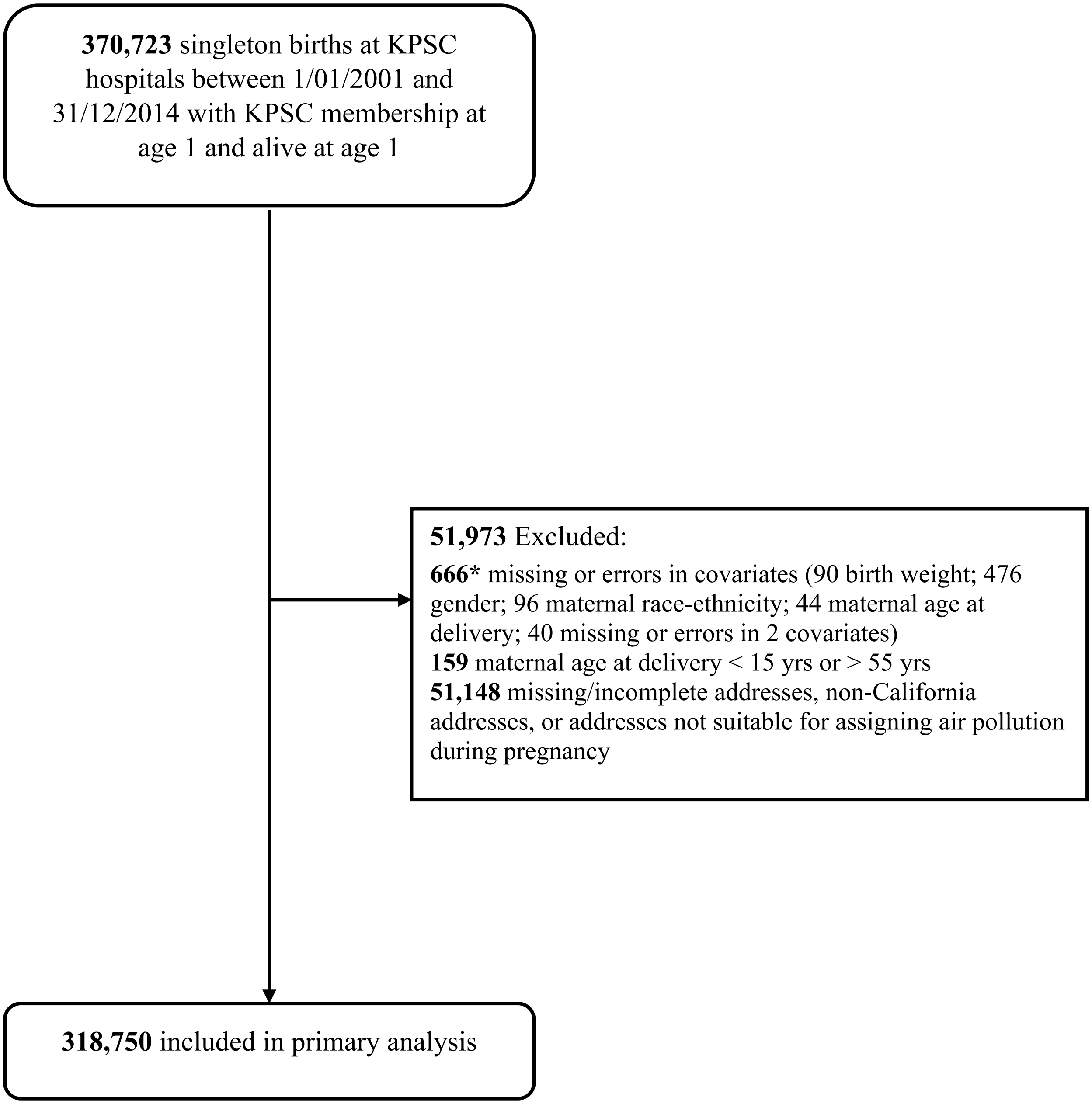

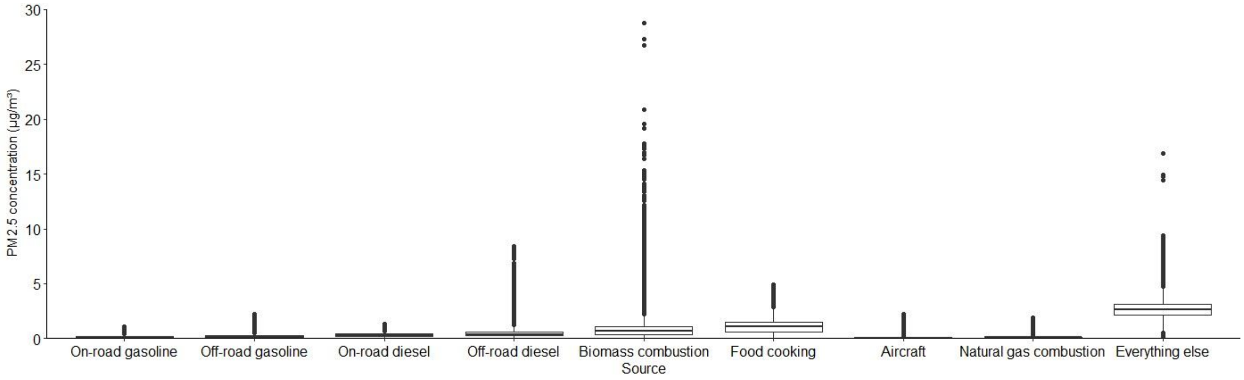

Figure S2. Pregnancy-averaged source-specific exposure concentrations across participants
